# In Vitro Methods of Assessing Protein Quality for Poultry

**DOI:** 10.3390/ani10040551

**Published:** 2020-03-25

**Authors:** Dervan D.S.L. Bryan, Henry L. Classen

**Affiliations:** Department of Animal and Poultry Science, University of Saskatchewan, Saskatoon, SK S7N5A8, Canada

**Keywords:** dietary protein, poultry, digestibility assay, in vitro, pH stat method, pepsin digestibility assay

## Abstract

**Simple Summary:**

Over the years, broiler chickens have been selected for rapid growth which makes them very efficient at depositing body protein in a short period of time. This is important since the broiler sector is expected to contribute to the growing global demand for poultry meat. In light of this, the quality of proteins fed to poultry is becoming more important. The concept of protein nutrition is based on the sequential process through which proteins are digested, and the amino acids are absorbed and become available for metabolic processes. The nutritional quality of protein ingredients for poultry is based on their amino acid bioavailability. Animal and plant ingredients are the main sources of protein used in poultry diets and they vary in digestibility and amino acid composition. Although in vivo digestibility assays for poultry are available, they are expensive and time consuming to conduct. In vivo digestibility assays are the optimum tools for characterizing protein sources to be used in commercial production, but it is not always practical to conduct these assays in commercial settings. Commercial production, therefore, relies on the use of other assays such as in vitro assays to evaluate the quality of protein sources.

**Abstract:**

Protein quality assessment of feed ingredients for poultry is often achieved using in vitro or in vivo testing. In vivo methods can be expensive and time consuming. Protein quality can also be evaluated using less expensive and time consuming chemical methods, termed in vitro. These techniques are used to improve the user’s efficiency when dealing with large sample numbers, and some mimic the physiological and chemical characteristics of the animal digestive system to which the ingredient will be fed. The pepsin digestibility test is the in vitro method of choice for quick evaluation of protein sample during quality control and in most research settings. Even though the pepsin digestibility test uses enzymes to liberate the amino acids from the protein, it does not mimic normal in vivo digestive conditions. The results obtained with this method may be misleading if the samples tested contain fats or carbohydrates which they often do. Multi-enzyme tests have been proposed to overcome the problem encountered when using the pepsin digestibility test. These tests use a combination of enzymes in one or multiple steps customized to simulate the digestive process of the animal. Multi enzyme assays can predict animal digestibility, but any inherent biological properties of the ingredients on the animal digestive tract will be lost.

## 1. Introduction

Over the years, broiler chickens have been selected for rapid growth which makes them very efficient at depositing body protein in a short period of time. This is important since the broiler sector is expected to contribute to the growing global demand for poultry meat. In light of this, the quality of proteins fed to poultry is becoming more important. Animal and plant ingredients are the main sources of protein used in poultry diets and they vary in digestibility and amino acid composition [1,2,3].

The concept of protein nutrition is based on the sequential process through which proteins are digested, and the amino acids are absorbed and become available for metabolic processes. The nutritional quality of protein ingredients for poultry is based on their amino acid bioavailability. Animal proteins are composed of twenty-two amino acids [4]. Ten of the twenty-two amino acids in poultry meat proteins cannot be synthesized in large enough quantity and, therefore, must be provided in the diet for proper growth and metabolic function [5]. 

Digestibility is used in practice as an estimator of the amino acid bioavailability in poultry diets [6]. Digestible protein is the proportion of protein that is digested and absorbed in the form of amino acids [6]. On the other hand, amino acid bioavailability is the proportion of an amino acid in a form that is suitable for protein synthesis after the protein has been digested and amino acids absorbed [7]. Since the 1990s, most poultry nutrition research used digestibility assays when evaluating protein feed ingredients instead of bioavailability [5], because they do not require the free form of the amino acid during the evaluation [7]. The digestibility coefficient obtained can be used directly by nutritionist during ration formulation [5]. 

Although in vivo digestibility assays for assessing protein quality for poultry are available, they are expensive and time consuming to conduct. In vivo digestibility assays are the optimum tool for characterizing protein sources to be used in commercial production, but it is not practical to conduct these assays in a commercial setting. Commercial production therefore, relies on the use of other assays such as in vitro assays to evaluate the quality of protein sources. The pros and cons of in vitro and in vivo assays are covered in the subsequent review. It was clear that there is a need for a poultry specific in vitro protein digestibility assay for assessing protein sources commonly fed to poultry. This review presents a critical overview of current in vitro protein digestibility assays relevant to poultry and the application of their methodology in assessing protein quality of ingredients for poultry. The objectives of this review paper were: (1) To provide a comprehensive review of the in vitro methods currently available which has the potential or has been applied in the assessment of protein quality for poultry, and (2) to explore potential methodological factors which might be important in the assessment protein digestion. 

## 2. Methods of Assessing Protein Quality 

Traditionally, protein quality is assessed by evaluating the extent to which amino acids are digested and absorbed from the ingredient. Estimation of protein digestibility is normally achieved by feeding the feed ingredient to the intended animal and assessing protein or amino acid digestibility. This technique is termed in vivo. Protein quality can also be evaluated using less expensive and time consuming in vitro chemical methods. These techniques are used to improve the level of precision while mimicking the physiological and chemical characteristics of the digestive system of the animal to which the ingredient will be feed. 

To obtain useful information on the digestibility of nutrients without the use of in vivo assays, researchers often employ the use of in vitro assays. In theory, in vitro digestibility assays should closely simulate the digestive process of the intended animal [8]. Depending on the nature of the research, it is expected that an intended in vitro assay should be reproducible, cheaper than available in vivo assays and simple to perform while giving fast results [9]. Methods for evaluating nutrient digestibility in vitro for simple stomach animals have been reviewed by others [8,10]. Only those methods applicable to protein digestion in poultry will be discussed. 

### 2.1. Chemical In Vitro Methods 

Evaluating protein quality using chemical method provides less precision than in vivo techniques but can be used as a routine quality control measure. In the chemical engineering literature, it was known as early as the 1930s that an alkali solution could extract up to 95% of the protein from plant meal sources [11]. In the late 1960s, Rinehart was one of the first to employ the protein solubility technique as a measure of protein quality of soybean meal in the poultry industry [12]. While working at Purina Mills Inc., Rinehart evaluated the suitability of protein from soybean meal derived from different processing systems using potassium hydroxide (KOH).

The ability to predict animal performance is one of the most important criteria of any chemical assay [13]. It was not until the 1950s that Lyman et al. [14] established a relationship between bird performance and the solubility of protein feed ingredients used in poultry diets. The study evaluated the correlation between a chick growth assay and the use of a protein solubility technique using sodium hydroxide as the alkali solution. In the solubility technique, one gram of cottonseed meal with four glass beads was placed in an Erlenmeyer flask with 100 mL of 0.02 N sodium hydroxide solution. The flask was agitated continuously at 37 °C for an h, and then the mixture centrifuged for 5 min at 3000× *g*. After centrifuging, the solution was filtered and aliquots evaluated for protein concentration [14]. 

The solubility index method was not adopted as a routine measure of protein quality in the poultry feed industry until the test was validated. A study was reported in which the protein solubility technique was used to evaluate soybean quality in poultry feed [12]. This study provided the foundation for the evaluation of protein quality using the solubility technique. The researcher [13] revived the technique when they proposed the use of sodium tetraborate at 40 °C as a more sensitive test for detecting changes in protein quality due to overcooking of meals. By the end of the late 1990s, protein solubility using KOH became a routine technique in research evaluating dietary protein [13,15,16,17]. Researchers used the protein solubility index to evaluate canola meal quality and found that the 0.5% sodium hydroxide assay did not accurately predict canola meal lysine digestibility in broiler chickens [18]. This suggests that the relationship between protein solubility and amino acid digestibility is ingredient specific.

Protein dispersibility index (PDI) is another method used to evaluate the quality of protein ingredients. This technique involves high speed mixing of a protein sample in water, followed by the assessment of solubility [17]. In the literature, PDI may be referred to as water dispersible protein or water-soluble protein [19]. In 1970, the PDI technique was published as two official and tentative methods of the American Oil Chemists Society [19]. Veltmann and coworkers [20] evaluated the quality of soybean meal used in poultry diets employing the PDI method. The PDI method was able to distinguish between normal processed meals and meal heat-treated to escape rumen degradation. In that same study, a chick growth assay showed that there was no difference between the bioavailability of the protein from the two meals [20]. This suggested that the PDI method did not correlate well to the bioavailability of protein from the ingredient tested.

In 1978, the American Oil Chemists Society published a revised PDI method, which was corrected in 1979 as method Ba 10-65. In brief, 20 g of protein is mixed for 10 min at 7800× *g* with 300 mL of water. A portion of the mixture is centrifuged and the nitrogen content of the solid fraction and the original protein sample measured [21]. The percent dispersed protein is calculated as the protein loss from the original sample to the water. Batal and coworkers [17] compared the revived PDI method against the urease index and KOH solubility test. Of the three tests, the PDI method was more effective and more sensitive in detecting the minimum adequate heat processing conditions required for soybean meal fed to chickens. 

Since the 1980s, the PDI method has become a routine technique used worldwide by researchers [17,20,22,23,24] to assess the quality of protein sources used in monogastric animal feeds. While chemical methods provide an overview of the protein quality of feed ingredients, they do not give a good indication of how much of the nutrient will be absorbed by the animal. Protein solubility index and PDI methods are used as measures of ingredient quality in most poultry nutritional research evaluating high protein ingredients. The information gained from the PDI method and protein solubility index does not provide useful information for diet formulation in a commercial setting, but they are often used in quality control programs.

### 2.2. pH-Stat/Drop Method

As protein samples are hydrolyzed by digestive enzymes, they release protons from the cleaved peptide bonds, which changes the pH of the reaction media [25]. In the early 1970s, Maga, Lorenz, and Onayemi evaluated the extent to which dietary protein undergoes proteolysis. They realized that there was a close relationship with the initial rate of hydrolysis of the proteins from 0 to 10 min and the digestibility of the protein samples. The rates of hydrolysis of the protein samples were evaluated as an indirect measure of the pH of the reaction mixture over time. In their system, the protein samples were incubated with trypsin at 37 °C in a water bath for 10 min while evaluating the pH change. However, this method lacked precision in predicting the bioavailability of protein [26,27]. 

To improve precision in predicting bioavailability with the Maga et al. [25] method, Vavak modified the above procedure in a master’s thesis while working with distiller’s grain protein concentrate [26]. During the modification of the procedure, various enzyme combinations were tested in an effort to gain improvement in the correlation coefficients between pH drop and protein digestibility in rats. The trypsin-chymotrypsin combination gave superior correlation coefficients compared to the initial single trypsin proposed by Maga et al. [25]. Hsu and coworkers [27] suggested that the methods presented by Maga et al. [25] and Vavak [26] were too time consuming and complicated for routine quality control. 

A faster method was developed, which could be completed in 1 h [27]. In this method, the trypsin-chymotrypsin enzyme combination was replaced with a multi-enzyme mixture composed of trypsin, chymotrypsin and peptidase. The correlation coefficient with the apparent digestibility of protein for rats was 0.9 using this new multi-enzyme system after evaluating 23 food protein sources. The method was also able to detect the effects of trypsin inhibitor, chlorogenic acid and heat processing on the digestibility of the protein tested. The pH drop method was susceptible to the buffering capacity of the protein source since high ash content affected the digestibility results [27]. A researcher used the pH drop method proposed by Hsu et al. [27] modified by Satterlee et al. [28] to evaluate various high protein feed ingredients while correlating the results to true digestibility in caecectomized cockerels [10]. There was a good correlation with lysine digestibility in caecectomized cockerels and the pH drop test across the ingredients tested. The test, however, showed no relationship to lysine digestibility and protein efficiency ratio in various qualities of feather meal and meat meal samples. 

To overcome the susceptibility of the pH drop test to the buffering capacity of protein samples, Pederson and Eggum revised the pH drop method proposed by Hsu et al. [27]. During revision, the consumption of alkali was used as an indirect measure of true protein digestibility values in rats. The pH of the reaction was held constant at 8 during titration with alkali over a 10 min period [29]. The correlation coefficient was improved from 0.9 [27] to 0.96 [29] with a residual error of 1.29 after evaluating 30 protein samples. The authors [29] suggested that the effects of ash content on the test results were due to differences in mineral content, which was mostly due to the influence of calcium. The authors proposed the use of two different regression equations to accurately predict the digestibility of protein samples from plant and animal origins. Using literature derived prediction equation for a specific kind of protein source was unreliable when using the pH-stat method [30]. To measure the degree of hydrolysis of protein, the method requires knowledge of the average dissociation of the α-amino groups of the protein sample and the number of peptide bonds present in the territory structure of the main proteins present in the ingredient [29].

Due to the limitations mentioned, the pH-stat test has been used mostly in food science research to predict the digestibility of highly digestible pure protein sources [29,30,31]. Such pure protein sources typically have data about the average dissociation of the α–amino groups and the number of peptide bonds present. Since the early 1990s, the pH-stat method has been used to evaluate only aquatic animal feed ingredients [32,33]. To address the limitations of the method, casein average dissociation constant and the number of peptide bonds were used as the standards when calculating the degree of hydrolysis [33]. So far, the data generated with the pH-stat method has been consistent with *in vivo* digestibility assays, especially with the use of purified enzymes extracted from the species to which the ingredient has been fed [32,33]. The pH-stat method has become a valuable tool for aquatic nutritional research, but not for terrestrial animals. The good digestibility correlations seen with aquatic species are probably due to the simple nature of their digestive tract and the use of highly digestible protein sources such as fish meal. 

### 2.3. Closed Enzymatic Methods

These systems are used to evaluate the digestibility of nutrients with multiple or single enzymes while simulating part or all of the in vivo digestive process [8]. The system is flexible, so the procedure and enzymes used may vary to meet the specific needs of the research objectives. Only those procedures used specifically to evaluate the digestibility of protein samples will be reviewed. The digestibility of protein is tied to the amino acid content and to the specificity of the digestive enzyme used to free them from complex peptides [34]. 

#### 2.3.1. Pepsin Assay

The pepsin digestibility assay is one of the most widely used assays to evaluate the quality of feed and protein ingredients. Gehrt and coworkers and Sheffner and coworkers were the first group of researchers to employ a single enzymatic method to evaluate the digestibility of protein using pepsin [35,36]. In their procedure, 1 g of protein was incubated with 25 mg of pepsin in 30 mL of 0.1 N sulfuric acid at 37 °C for 24 h, during this time, the samples were stirred intermittently [35]. After incubation, the samples were placed in a boiling water bath for 10 min. Samples were cooled and the pH adjusted to 2 followed by the addition of one volume each of 10% sodium tungstate and 2/3 N sulfuric acid. The mixtures were filtered after standing for 10 min, and then the filtrate adjusted to pH 6.8 and analyzed for amino acids. When the digestibility data were regressed against the biological value of the protein samples for rats, there was a 0.998 correlation [36].

The pepsin digestibility assay was not accepted as a routine protein quality evaluation until 1959. The Association of Official Analytical Chemists (AOAC) adopted a revived version of the method proposed by Gehrt et al. and Sheffner et al. [35,36]. Hydrochloric acid was used instead of sulfuric acid, and all the fat was extracted from the samples using ether before digestion. The sodium tungstate and pH steps were eliminated. In 1972, the procedure was revised to improve the filtration step and the pepsin concentration was defined as 0.2%. 

Since the 1959 AOAC publication of the pepsin digestibility method, it has been used extensively to evaluate high protein feed ingredient quality of both plant and animal origin [15,37]. Johnston and coworkers were one of the first group of researchers to use this method to evaluate poultry feed ingredients of animal origin [37]. After evaluating 20 commercial animal by-product meals, they were able to get a 0.91 correlation with the net protein utilization and the protein efficiency ratio for chickens. The pepsin digestibility procedure proposed by Johnson et al. [37], adjusted the pepsin concentration to 0.002% while eliminating the preliminary grinding and defatting steps. 

In another study, the same group of researchers evaluated various levels of pepsin in order to find a suitable level for use in the assay during routine evaluation of meat and bone meal samples fed to poultry [38]. Lower levels of pepsin (0.002%) were able to detect differences between the quality of the meat and bone meal samples, which was contrary to that of the AOAC 0.2% pepsin. Parsons and coworkers did a comparative study on the ability of 0.2%, 0.002%, and 0.0002% pepsin to detect differences in quality among 14 meat and bone meal samples [1]. They confirmed the findings of Johnson et al. [38] that the 0.002% pepsin level gave the best correlation with lysine digestibility in chickens. 

#### 2.3.2. Pancreatin

Some testing systems involve the use of pancreatin as the only enzyme source to digest protein samples. Riesen and coworkers described a single enzymatic method that used pancreatin to evaluate the quality of soybean meal in poultry [39]. The samples were ground in a power-driven mortar, 100 mg or 300 mg of pancreatin was added to 2 g of the ground samples in 50 mL of 0.2 M disodium phosphate buffer at pH 8.3. One mL of toluene was added to the solution, and the mixture incubated for 5 d or 12 h at 37 °C. At the end of each digestion period, the samples were heated with steam for 15 min to facilitate enzyme deactivation. The pH of the mixture was adjusted with glacial acetic acid to precipitate the undigested proteins. This method was able to detect the difference between overheated and the normal heated meals, but not the difference between the normal and under heated soybean meals. 

Ingram and coworkers modified the procedure by adding 1.2 g of pancreatin to 12 g of sample in 300 mL of buffer for 6 h [40]. The pattern of amino acid released from the samples correlated with the growth of chickens fed the same samples of soybean meal [40]. In another study by Anwar [41], the pancreatin in vitro test was used to evaluate the quality of cottonseed meal, groundnut meal, meat meal and fish meal [41]. The method was not reliable for fish meal and groundnut meal but gave fair results for meat meals. The one-step pancreatin method has been used routinely by many food scientists to evaluate the digestibility of various protein foods, but not by poultry nutritionists [42]. 

Pancreatic digestion is controlled by substrate concentration in vivo. An increase in protein concentration will promote an increase in proteolytic enzyme secretion [8]. In vitro digestibility methods using pancreatin as the only enzyme, source keeps the enzyme concentration constant when evaluating a range of protein sources [41]. However, the method lacks precession when evaluating a variety of protein sources [41]. Other researchers have found no difference between in vivo chicken ileal digestibility and the pepsin or pancreatin assay when ranking feather meal digestibility [43].

#### 2.3.3. Multi-Enzymatic Assays

A multi-enzyme method may use two or more enzymes while simulating one, two or all stages of the digestive process [8]. Multi-enzyme methods are more comparable to in vivo conditions since many enzymes are involved in the digestion of proteins. The digestion of proteins starts in the stomach under the action of pepsin and hydrochloric acid. The partially digested protein enters the small intestine where they are hydrolyzed by trypsin, chymotrypsin, elastase and carboxypeptidases [8].

In 1964, Akeson and Stahmann described a method using pepsin and pancreatin as enzyme sources. The method was developed to evaluate large numbers of food protein samples while reducing the labour load associated with the pepsin digestibility assay [44]. The method involved incubating 100 mg of protein sample with 1.5 mg pepsin in 15 mL 0.1 N hydrochloric acid for 3 h at 37 °C [44]. The reaction was neutralized with 7.5 mL of 0.2 N sodium hydroxide solution and then 4 mg pancreatin dissolved in 7.5 mL phosphate buffer with pH 8 was added. Fifty parts per million merthiolate were added to the mixture, which was incubated at 37 °C for 24 h. Samples of the digestion mixture were precipitated with acid and centrifuged at 1000× *g* for 30 min after which the supernatant was analyzed for amino acids. 

In 1973, Saunders and coworkers described a two enzyme system using pepsin and trypsin. The test occurred in a closed system using centrifuge tubes containing 1 g of protein sample suspended in 20 mL of 0.1 N hydrochloric acid and then mixed with 50 mg pepsin dissolved in 1 mL 0.01 N hydrochloric acid [45]. The mixture was incubated at 37 °C while gently shaken for 48 h, centrifuged at 20,000× *g* for 5 min, and the supernatant removed. The solid was suspended in 10 mL water and 10 mL of 0.1 M phosphate buffer with pH 8 and 5 mg of dissolved trypsin. The mixture was incubated at 23 °C for another 12 h then centrifuged and the solids washed with 30 mL of water five times, with centrifuging and removal of the supernatant each time. The solid was filtered through a 1.2 µm Millipore filter, air-dried, and analyzed for amino acids. 

Both the pepsin-pancreatin and pepsin-trypsin methods were able to give good correlation between the in vivo digestibility values for various food proteins using rats [44,45]. The pepsin-pancreatin assay is known to give good correlation with amino acid digestibility of 0.84 in cereal gains with true amino acid availability in chickens but was less reliable for soybean meal and corn gluten meal [46]. However, the pepsin-pancreatin test gave an excellent correlation of 0.91 between the in vivo ileal digestibility of protein of 15 feedstuffs in pigs [47]. The test proposed by Saunders et al. [45] has been used to some extent to evaluate protein digestibility in poultry [48,49,50].

Dialysis cell method is a non-static system in which products of digestion are removed from the substrate as they become available. When simulating in vivo protein digestion with in vitro techniques, the rate of hydrolysis may be compromised by the accumulation of end products in the system [46,51]. The rate of hydrolysis can be improved if the digestion products are removed from the system as digestion occurs [46]. To prevent the inhibition of proteolysis by the end products, dialysis has been proposed to remove digestion products [52,53]. They conducted their experiments in dialysis bags to facilitate the removal of the end products during incubation of the protein source with the enzymes. 

In 1982, Gauthier and coworkers et al. adopted the dialysis principle of [52,53] and presented a method in which the dialysis solution was continually replaced as the incubation proceeded [34]. The content of the dialysis bag was stirred constantly during the digestion process. In brief, 400 mg nitrogen (6.25 × %N) of protein was suspended in a beaker with 100 mL of 0.1 N hydrochloric acid. The beaker was shaken and placed in a water bath at 37 °C for 30 min. The pH of the solution was adjusted to 1.9 then 20 mL of solution containing 5 mg pepsin per mL of 0.1N hydrochloric acid added. The mixture was incubated for 30 min, the pH was adjusted to 8 and transferred to a dialysis bag with a 1000 Da molecular weight cut off. The bag was placed in a U-shaped container with inlets from a peristaltic pump and outlets to a beaker. Twenty mL of a solution containing 5 mg pancreatin per mL sodium phosphate buffer was added to the dialysis bag, which was continuously washed with 37 °C sodium phosphate buffer at a flow rate of 212 mL/h. Samples of dialysate were collected at different time intervals and analyzed. The method was able to detect the effects of heat and alkali treatment on protein digestibility in foods [34]. 

The digestion unit size plus the use of handmade apparatus were limitations for its use in routine protein evaluation [54]. Savoie and Gauthier modified the design presented by Gauthier et al. [34]. The improvements included the use of a magnetic stir bar and the construction of a cell with an inner compartment fitted with a dialysis membrane. The cell was 100 mm long in comparison to the 298 mm original unit. There was free access to the reaction chamber without disruption of the reaction. Each cell was designed to work as a single unit or part of the multi-unit system. The system developed was very flexible and could be used to measure the release of any product from enzymatic hydrolysis [54].

The dialysis cell method has been applied to study protein digestibility across a number of disciplines [55,56,57]. This method was able to identify differences in the rate of release of amino acids from different sea bream feed samples [57]. The system was flexible to accommodate the use of crude enzyme extract from sea bream as the digestive enzyme. A comparison between the pH-stat and the dialysis cell method showed that the dialysis cell method was able to identify which products were released from the protein as well as the digestion kinetics of the protein samples [55]. The effects of different processing methods on the digestibility of legume proteins were identified with the dialysis cell method [56]. A detailed description of the availability of different amino acids and the rate at which they were released during digestion was obtained from different protein sources [56,58]. The main disadvantages of the dialysis cell method are the complexity and the number of samples which can be digested in a given run. This method uses custom made dialysis cells, peristalsis pumps, and fraction collectors which can be expensive. Savoie and Gauthier recommend that no more than 6 cells should be used simultaneously due to the manual inputs needed. From a practical point of view, an in vitro method must be simple and easy to implement for it to be adopted by poultry nutritionist [54].

## 3. Factors Influencing Protein Digestion

The digestibility data obtained by in vitro methods vary even within the same method for the same ingredient. This variation may be due to a number of issues associated with in vitro digestibility systems. Enzymes and their concentration seemed to be one of the most important factors influencing in vitro digestion [1,26,59]. The specificity of enzymes and their ratio to the substrate will determine the level of hydrolysis achieved [8,59]. 

### 3.1. Enzyme Specificity

Table 1 shows a list of enzymes involved in protein digestion. The first enzyme responsible for the initiation of protein digestion in poultry is pepsin [8]. This enzyme will only cleave the N-terminal of aromatic amino acids like tyrosine, tryptophan and phenylalanine [4] at low pH. Hydrolysis by pepsin results in smaller peptides that enter the duodenum for further hydrolysis by pancreatic protease [8]. As suggested by Assoumani and Nguyen [10], trypsin will only break a lysyl or arginyl peptide bonds to expose lysine or arginine terminal residues at basic pH. Trypsin binds only to the positive side group of arginine and lysine, where the peptide is cleaved at those amino acids [4]. 

The ability of enzymes to hydrolyze substrate may depend on the presence of other enzymes. The activation of chymotrypsin is dependent on the presence of trypsin [4]. Chymotrypsin will act on proteins and peptides, but will also hydrolyze esters and amides [60]. Chymotrypsin cleaves peptides over a wider range of sites than trypsin, both aromatic and hydrophobic side chains of amino acids residues [4]. Peptide bonds involving tyrosine, tryptophan, phenylalanine and glutamyl, leucyl, asparaginyl residues are cleaved by chymotrypsin [4,8].

Lysine or arginine are released from small peptides by carboxypeptidase-B, which is specific for C-terminal basic groups [8]. Animal protein meals may contain high levels of collagen due to the nature of the type of rendering material. Digestion of this meal in vitro may need additional collagenase enzymes during the pancreatic digestion stage [61]. Bonds hydrolyzed in protein feed samples are enzyme-specific, so in vitro digestion models should take this into account by using multiple enzymes [8]. 

### 3.2. Protein Structure and Forms

The structure of the protein samples and the food matrix in which the samples are presented will influence protein in vitro digestibility [10]. Protein feed ingredients may contain free amino acids, peptides of various lengths, secondary structure proteins (α-helix, β-pleated sheets, β-turns and superhelix), tertiary structure proteins and quaternary structure proteins [4]. Secondary structure proteins such as scleroproteins, which include collagen, elastin, and keratin, are poorly digested in simple stomach animals [62]. Protein sources containing high levels of these proteins will have limited bioavailability. Higher protein structural configuration requires more time and higher enzyme concentration to achieve greater hydrolysis [4,62]. 

Secondary structure proteins resist digestion due to the nature of their individual structures. Feather meal, for example, contains high levels of keratin [43], which has highly cross-linked disulphide bonds along the pleated sheet configuration [62]. This makes the protein almost insoluble in water and thereby reduces the action of pepsin and subsequent pancreatic actions [43]. Samples of meat and bone meal may contain elastin and collagen after being produced from tendons, ligaments and bone scraps of animals. Elastin and collagen also contain cross-linking in their helix structures which may influence digestion [62]. 

The matrix in which the protein is presented in the protein source may limit the access of proteolytic enzymes. Plant proteins are often presented in a matrix with cell walls, lipids, and complex sugars, and may also be organized into specialized storage vacuoles [10,62]. The ability of proteolytic enzymes to access those proteins may depend on the ability of other enzymes to free protein from the matrices [8]. The digestion of protein from plant sources in monogastric animals is closely linked to the protein associated with plant cell wall components [63]. Non-starch polysaccharides are known to protect proteins from enzymatic digestion in a variety of plant feed ingredients in poultry [64]. Solubilization of the cell wall components of plant-sourced protein meals with various carbohydrase enzymes were able to improve the availability of the protein to chickens [63,64].

### 3.3. Enzyme Activity

In vitro digestion may be influenced by the activity of the enzymes used while enzyme activity is affected by factors such as pH, temperature, ratio of enzyme to substrate, and incubation time [8]. As proteins are hydrolyzed by enzyme in vitro, the pH of the mixture will be reduced by the release of protons from the cleaved peptide bonds [25]. If the original pH of the reaction moisture is further away from the optimum pH of the enzyme, the rate of hydrolysis will be reduced drastically in a short period of time. In the pH-stat method, pH is held constant in the optimal range for the enzyme via automated alkali titration [29]. To achieve optimal reaction conditions, most in vitro assays select appropriate starting pH for the enzyme used [10]. The pepsin digestibility assay requires an acidic condition [36], while the pancreatin assay requires a basic environment [39].

The temperature may play a regulatory role as it relates to enzyme activity. Like all chemical reactions, temperature increases the amount of kinetic energy and increases the velocity at which molecules collide in an enzymatic reaction [4]. In vitro digestibility assays using protease keep the temperature of their reaction between 37 and 45 °C [8]. Enzymes are proteins and all proteins can be denatured at high temperatures; therefore the optimal temperature for a given enzyme is always close to the body temperature of the organism from which the enzyme was derived [4]. In vitro assays should reflect in vivo conditions so the temperature at which the reaction takes place is often that of the animal’s internal temperature [33,47].

The ratio of enzyme to substrate and the incubation time varies across individual in vitro assays [1,8,51,59]. Generally, the incubation time can range from 0.5 to 45 h depending on the kind of in vitro assay [10]. The enzyme to substrate ratio is often a function of the specific activity of the enzyme. The specific activity of an enzyme is often defined as the amount of product produced from a specific substrate over time while maintaining the reaction at a fixed pH and temperature range [4]. Enzymes from different preparations with different specific activities are often used for the same in vitro assay [54,58]. The ratio of pepsin used with 4 mg nitrogen of sample in the dialysis method ranged from 5 to 7 mg/mL pepsin [34,54]. Pepsin concentration used in the pepsin digestibility test ranged from 0.02 to 2.5 g/L and the sample size of the protein may be expressed as g of nitrogen per sample [8]. To avoid confusion in the literature, an in vitro method should define the enzyme to substrate ratio and the specific activity of each enzyme in the assay [65].

### 3.4. Anti Nutritive Agents of Test Samples

Anti-nutritional compounds are often secondary metabolites and structural components of plants that interfere with the metabolic activities of animals when present in feed ingredients [66]. These compounds provide structural support and some metabolites have evolved into defence chemicals to protect plants from insect damage [67]. Some anti-nutritional compounds represent important storage minerals and intermediate molecules used in various pathways by the plant [66]. The main action of these compounds tends to disrupt the digestive process via multiple modes of action. 

#### 3.4.1. Sinapine and Tannins

A phenolic compound found in many plant feed ingredients is sinapine, which is a choline ester derived from 3, 5-dimethoxy-4-hydroxyinnamic acid or tannins [68]. Growing plants use sinapine as their main source of sinapic acid and choline [69]. High levels of sinapic acid can react with other compounds to create a colour change and produce a bitter taste in plant feed ingredients [70]. During oxidation, phenolic acids may react with proteins to form indigestible complexes like quinines which bind to the functional group of lysine and methionine [68].

Tannins are another set of water soluble polyphenolic compounds that may be found in protein meals of plant origin [71]. They are normally present in legume seeds, cereal grains, and oilseeds [68,72]. Tannins are generally grouped into hydrolyzable and condensed tannins. Hydrolyzable tannins may have esters of gallic, m-digallic, or hexahydroxydiphenic acids, which are easily hydrolyzed [71]. Condensed tannins resist hydrolysis and are polymers of flavan-2, 4-diol and flavan-3-ol or a mixture of both [72]. Tannins precipitate protein out of solution through the formation of soluble and insoluble complexes [68], and are known to reduce the digestibility of amino acids in poultry [73]. Tannins inhibit the absorption of protein from the digestive tract [72,73]. Low molecular weight tannins may be absorbed from the intestine and cause toxicity through the inhibition of key metabolic pathways [72,73].

#### 3.4.2. Protease Inhibitors

Almost all plant protein sources available for use in animal production contain some type of protease inhibitor [74]. Even commonly consumed foods such as legumes, cereal grains, and tomatoes contain protease inhibitors [72]. Protease inhibitors block the activity of trypsin, chymotrypsin [62], elastase, and carboxypeptidase [75]. Trypsin inhibitor can be found in field pea, peanut, wheat, soybean, rapeseed, lupin, and sunflower seeds [62,75]. 

Of the plant protein sources used in poultry production, soybean is generally considered to have the highest trypsin inhibitor activity [72]. The inhibitors bind to the active site of the enzyme, thereby reducing their ability to lower the kinetic energy needed during proteolytic cleavage [4]. The two main inhibitors found in soybean are from the Kunitz and Bowman-Birk inhibitor families [62]. Kunitz is about 21.4 kDa with high affinity for trypsin, while Bowman-Birk is about 8 kDa and has high affinity for both trypsin and chymotrypsin [72]. 

When birds were fed diets containing raw soybean, the granules of the pancreatic acini were totally depleted in 2 h after feeding and the size of the pancreas increased after 8 d [76]. The pancreatic activity of the birds at 16 d was twice the activity before they were given the diet and the birds growth was reduced drastically. Protease inhibitor activities can be reduced through various heat processes, but complete elimination is often not possible in commercial soybean products [15,74].

#### 3.4.3. Phytate

Feed ingredients derived from plants contain some level of phosphorus stored as phytic acid or phytate which are also known as myo-inositol hexaphosphoric acid and myo-inositol hexaphosphate respectively [77]. Phytate is predominantly found in the seeds of plants, which makes animal feed derived from oilseeds and cereal grains a source of phytate [78]. During germination, the inorganic phytate is hydrolyzed by enzymes to produce phosphate which the plant uses for its growth [79]. Phytic acid has strong mineral binding capacity through its six phosphate groups, which actively bind zinc, iron, calcium, and magnesium [79]. Phytate’s chelating ability results in complexes with nutrients such as proteins and minerals [80]. 

The anti-nutritional effects of phytic acid on protein digestion can occur via direct or indirect modes of action. During protein digestion, phytate may bind to metal cofactors needed for the activity of aminopeptidases and carboxypeptidases [4,72]. Phytate may also bind with protein to form complexes in acidic and neutral pH conditions [80], which may inhibit the activities of digestive enzymes [81]. Intestinal phytase activity observed in poultry [82] may depend on magnesium as a cofactor. In such a case intestinal phytase may not be able to hydrolyze a substantial amount of the dietary phytate if sufficient magnesium is not present. However, in practical feeding situation the poultry industry has incorporated exogenous phytase in poultry diets [83]. The exogenous phytase hydrolyzes the ester bond between the inositol ring and phosphate group, thereby releasing phosphorus and reducing the anti-nutritive effects on protein digestibility. This elicits a question of whether exogenous phytase enzymes should be part of an in vitro protein digestibility assay for poultry. 

#### 3.4.4. Effects of Ingredient Processing

Proteins used in animal production are often by-products of other processing industries. The nutritional quality of these proteins is a function of the processes used in meal production. Plant-based protein sources generally will contain some form of anti-nutrient and thus require processing to reduce their effects when fed to animals. Protein meals of animal origin are waste products from food processing facilities. As such, the raw materials may contain higher levels of microbial contamination and require additional processing before it is fed to animals. 

The major anti-nutritional compounds found in plant-based protein sources can be reduced through some form of heat treatment. Unfortunately, amino acid digestibility in chickens may be compromised if the heat treatment used is excessive [12] or not enough [22]. Autoclaving flaxseed at 120 °C for 20, 40, and 60 min resulted in changes in the α-helix to β-sheet ratio of the protein fraction [84]. Rumen degradable protein is reduced with increased autoclaving time which suggested that the protein resisted digestion as a result of the change in α-helix to β-sheet ratio. This would be true if that same protein was fed to non-ruminants and the effects would be more severe.

During the commercial production of canola meal using the prepress-solvent extraction system, the meal is subjected to toasting during hexane removal [18]. Amino acid digestibility and the content of the meal are reduced after toasting. The elimination of the spurge steam during toasting could alleviate the loss of amino acids [18]. Soybean meal production involves solvent extraction as well. Ideally, the soybean is exposed to 105 °C for half h [85], but if the meal is heated to 121 °C, the concentration and digestibility of amino acids, especially lysine, are reduced [86]. The loss of amino acids during the production of meals from the solvent extraction process may result in poor growth in chickens fed meals processed under such conditions [13,87]. 

Amino acid loss during heating processing of protein meal may involve Maillard reactions, were a sugar-amine complex is formed from the reaction of sugars and ketones with amino acids, proteins, and peptides in food [88]. Mauron suggested that Maillard reactions involve early, advanced, and final stage reactions. Early Maillard reaction involves a reversible condensation of the carbonyl group of the sugar with the amino group of the amino acid, peptide, or protein to form a hydrolyzable N-substituted glycosylamine and then 1-amino-1-deoxy-2-ketose. At the early stage, food does not have any browning or flavour, but its nutritive value is reduced. During the advanced stage of the reaction, amines are released and are used as catalysts in reactions to form intermediate flavour products such as acetaldehyde and pyruvaldehyde [89]. The final reaction produces a dark brown nitrogen-containing pigment composed of decomposed amino acids, heterocyclic amines, melanoidin polymers and aldol condensation products [88]. 

The stages of the Maillard reaction requires specific reaction conditions to be successful [88]. Temperature and moisture are the two important parameters which govern each stage of the Maillard reaction [88]. Experimental simulations of Maillard reaction generally take place in solutions and the formation of melanoidin polymers is an exponential function of heating [90]. Reactions of D-xylose and glycine in aqueous solution at 22, 68, and 100 °C produce a temperature-dependent increase in aromaticity or high molecular weight melanoidin polymers [90]. The rate of the Maillard reaction is defined as the function Q_10_ which is the increase in rate for every 10 °C. As the temperature increases from 22 to 100 °C the quantity of high molecular weight melanoidin increases and the low soluble intermediate products of the Maillard reaction decrease [90]. 

Protein meals of animal origin do not contain the high levels of sugars found in meals of plant origin, so are less likely to undergo Maillard reaction when exposed to heat treatment. The natural soluble carbohydrate concentration of dried animal protein meals range from 0.3 to 1.3% [91], which is far less than what would normally be present in plant-based meals [13,87]. The meals are prone to Maillard reaction if they are exposed to soluble carbohydrate during autoclaving which has been shown to reduce meal digestibility [91].

Large amounts of meat and bone meal are produced by the rendering industry, but the quality of those meals can vary [37]. The variability in the quality of meat and bone meal can limit its use in poultry production [1]. Oxidation and enzymatic denaturing may occur depending on location and source of the raw material used in the rendering process. Polyunsaturated fats are known to react with atmospheric oxygen which results in the production of peroxides and other auto-oxidation products [92]. If the meal is kept in warm conditions, this could increase the formation of peroxides and secondary oxidation products. The application of heat in the presence of oxygen and polyunsaturated fats is known to increase the production of peroxides and secondary oxidation products [92]. This could be a factor during rendering if parameters such a temperature, time, and raw material polyunsaturated fat content are not controlled during meal production.

## 4. In Vitro Digestibility Systems Validation

**Figure 1:**
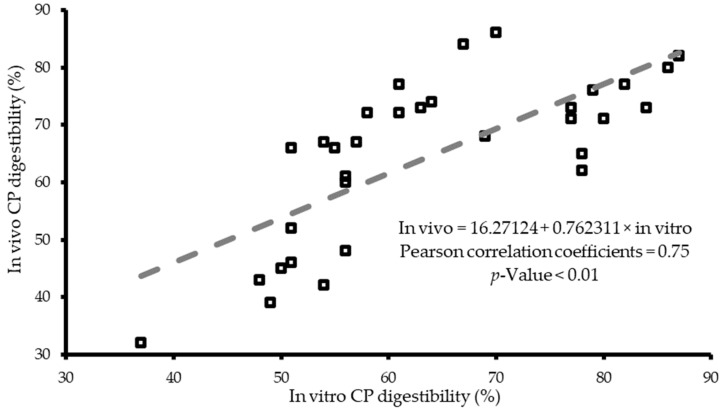
Plot of correlation between in vivo and in vitro crude protein (CP) digestible of nine high protein poultry feed ingredients [93].

One major challenge often encountered when developing in vitro models to evaluate protein digestion is the ability of a single model to effectively assay multiple kinds of feed ingredients. Due to this challenge, multiple quality control assays such as those based on the physicochemical properties of ingredients have been developed to help the feed industry. The in vitro model developed by Bryan et al. [59] evaluated nine different protein sources which are known to have variable digestibility and physicochemical properties. Correlation analysis between the PDI and KOH solubility of the ingredients and in vitro extent of crude protein (CP) digestion were all significant, with correlation coefficients (r) of 0.64 and 0.84, respectively. There was no correlation between the in vitro CP digestibility and the reactive lysine assay. This might be an indication that the reactive lysine assay was not a useful physiochemical candidate assay to compare with the developed in vitro assay. In Figure 1, such correlation with in vivo data is generally used as the last step for validating in vitro systems [93]. It is a common practice to conduct correction analysis in such circumstances but the validation of in vitro digestibility systems requires more analysis. 

In order to know if in vitro CP digestibility data are representative of the in vivo amino acid digestibility, regression and correlation analysis together (Table 2) were performed between the two sets of data generated by Bryan et al. [94,95]. The in vitro CP digestibility was positively correlated with all amino acids except for cysteine (CYS), which had a regression estimate P-value of 0.1. The correlation coefficients ranged from 0.43 to 0.71, except for CYS which was 0.30. The data presented in Table 2 shows the complexity of factors that might affect the interpatient of correlation validation of in vitro systems to in vivo. The in vitro model was developed using soybean meal (SBM) as the model protein source which has both pros and cons. Using SBM might have put the other ingredients at a slight disadvantage since the method optimized SBM digestibility for each stage of digestion and not the other meals. This could have accounted for some of the variation seen in the correlation coefficients of the amino acid with the in vitro CP digestibility. Based on the data presented in Table 2, the in vitro CP digestibility can be used as a predictor of in vivo amino acid digestibility; however, the correlation coefficients varied among amino acids so more samples need to be tested to form stronger prediction equations. 

**Figure 2:**
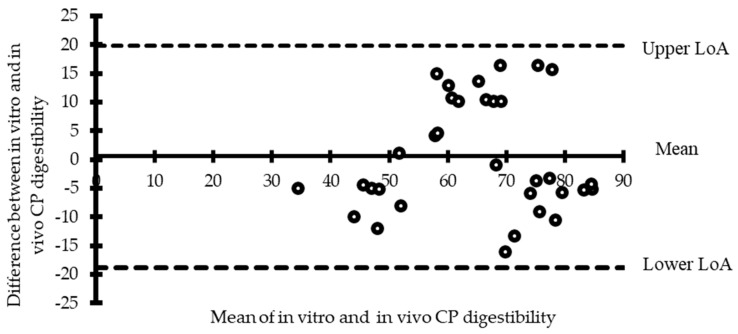
Bland Altman Plot of the difference between in vivo and in vitro crude protein (CP) digestible of nine high protein feed ingredients [93]; LoA: limits of agreement

Another approach in the validation step is to add more analysis. A one sample T-Test was performed comparing the difference between the in vitro and in vivo CP digestibility data of Figure 1 to a mean of 0 to see if there were differences between the two methods of assessing CP digestibility. This comparison suggests that there is no difference between in vivo and in vitro CP extent of digestion for the meals evaluated. The Bland Altman plot of the data presented in Figure 2 shows that there was no proportional bias between in vitro and in vivo CP digestibility data for any of the nine meals evaluated and all the data points collected during the assay fell in the 95% confidence limit. 

This indicates that the in vivo and the in vitro CP digestible data were in agreement for the digestibility of nine meals. Based on the correlation, the T-Test, and the Bland Altman plot results, the in vitro assay was able to predict the in vivo CP digestibility of the ingredients. The in vitro assay could, therefore, serve as a tool for assaying CP digestible of meals for broiler chickens. 

## 5. Conclusions

Protein quality assessment of feed ingredients for poultry is often achieved using in vitro or in vivo testing. The disadvantages associated with in vivo methods lead to the commercial acceptance of in vitro methods as the gold standard for assessing protein quality. These techniques are used to improve the user’s efficiency when dealing with large numbers of sample and some mimic the physiological and chemical characteristics of the animal digestive system to which the ingredient will be fed. Despite all of the advantages of these in vitro methods, they do not give a true replication of normal in vivo digestive conditions. This is because of the inability of those methods to mimic numerous biological factors involved in in vivo digestion and the complex interaction which exists with various ingredients. Multi enzyme assays can predict animal digestibility of proteins if they are designed properly. However, any inherent biological properties of the ingredients which might impact the animal digestive tract will be lost. Users of in vitro digestibility data should be aware of these disadvantages and take the necessary steps to validate in vitro methods and their data. In any case, in vitro digestibility methods are just estimates of in vivo digestion, which serve as a substitute in situations where in vivo digestion is not possible. 

## Figures and Tables

**Table 1:** Enzyme and their specific bond cleavage preferences.

Enzymes	Bond Cleave	Reference
Pepsin	N-terminal of aromatic amino acids phenylalanine, tryptophan and tyrosine	[4,8]
Trypsin	Lysyl or arginyl peptide bond to expose lysine or arginine	[10]
Chymotrypsin	Aromatic or large hydrophobic amino acid residues such as tyrosine, phenylalanine, tryptophan, leucyl, methionyl, asparaginyl, and glutamyl	[4,8,60]
Elastase	Glycine and alanine of elastin	[4,8]
Carboxypeptidase A	Peptide bond adjacent to the C-terminal end of a polypeptide chain,	[4,8]
Carboxypeptidase B	Basic amino acids from the C-terminal end of polypeptide chains	[8]
Collagenase	Alpha peptides and hydrogen bonds in the superhelix of tropocollagen and collagen	[61]

**Table 2:** Simple linear regression and Pearson correlation of in vitro digestible crude protein (CP) and in vivo standardized ileal amino acids digestibility of the nine meal samples [93].

Item	Regression Coefficients		ANOVA		In Vitro Digestible CP	
	Intercept	In Vitro Digestible CP	R^2^	MSE	Correlation Coefficients	*P*-Value
Aspartic acid	3.27	0.83	0.35	252.55	0.59	<0.01
Estimate SE	13.68	0.21	-	-	-	-
Estimate *p*-Value	0.81	<0.01	-	-	-	-
Threonine	27.35	0.55	0.35	115.73	0.59	<0.01
Estimate SE	9.25	0.14	-	-	-	-
Estimate *p*-Value	<0.01	<0.01	-	-	-	-
Serine	38.29	0.43	0.18	168.08	0.43	0.02
Estimate SE	11.15	0.17	-	-	-	-
Estimate *p*-Value	<0.01	0.02	-	-	-	-
Glutamic acid	22.19	0.75	0.50	112.11	0.71	<0.01
Estimate SE	9.11	0.14	-	-	-	-
Estimate *p*-Value	0.02	<0.01	-	-	-	-
Proline	13.72	0.75	0.35	206.60	0.59	<0.01
Estimate SE	12.36	0.19	-	-	-	-
Estimate *p*-Value	0.28	<0.01	-	-	-	-
Glycine	41.58	0.40	0.25	94.69	0.50	<0.01
Estimate SE	8.37	0.13	-	-	-	-
Estimate *p*-Value	<0.01	<0.01	-	-	-	-
Alanine	35.01	0.56	0.39	95.38	0.63	<0.01
Estimate SE	8.40	0.13	-	-	-	-
Estimate *p*-Value	<0.01	0.56	-	-	-	-
Cysteine	26.18	0.41	0.09	326.99	0.30	0.09
Estimate SE	15.55	0.23	-	-	-	-
Estimate *p*-Value	0.10	0.09	-	-	-	-
Valine	42.74	0.40	0.21	113.32	0.46	<0.01
Estimate SE	9.16	0.14	-	-	-	-
Estimate *p*-Value	<0.01	<0.01	-	-	-	-
Methionine	32.256	0.63	0.45	97.07	0.67	<0.01
Estimate SE	8.47	0.13	-	-	-	-
Estimate *p*-Value	<0.01	<0.01	-	-	-	-
Isoleucine	43.74	0.44	0.26	110.68	0.51	<0.01
Estimate SE	9.05	0.14	-	-	-	-
Estimate *p*-Value	<0.01	<0.01	-	-	-	-
Leucine	35.38	0.56	0.35	113.76	0.59	<0.01
Estimate SE	9.17	0.14	-	-	-	-
Estimate *p*-Value	<0.01	<0.01	-	-	-	-
Tyrosine	28.97	0.63	0.39	121.17	0.62	<0.01
Estimate SE	9.47	0.14	-	-	-	-
Estimate P-Value	<0.01	<0.01	-	-	-	-
Phenylalanine	39.97	0.5	0.29	120.73	0.54	<0.01
Estimate SE	9.45	0.14	-	-	-	-
Estimate P-Value	<0.01	<0.01	-	-	-	-
Lysine	34.44	0.57	0.50	62.50	0.71	<0.01
Estimate SE	6.80	0.10	-	-	-	-
Estimate *p*-Value	<0.01	<0.01	-	-	-	-
Histidine	12.17	0.84	0.48	150.04	0.70	<0.01
Estimate SE	10.54	0.16	-	-	-	-
Estimate *p*-Value	0.26	<0.01	-	-	-	-
Arginine	33.27	0.63	0.40	119.31	0.63	<0.01
Estimate SE	9.39	0.14	-	-	-	-
Estimate *p*-Value	<0.01	<0.01	-	-	-	-

R^2^: R-squared (variance for a dependent variable explained by variables in the regression model); MSE: Means square error; SE: Standard error.
